# Activation of CB2 receptors as a potential therapeutic target for migraine: evaluation in an animal model

**DOI:** 10.1186/1129-2377-15-14

**Published:** 2014-03-17

**Authors:** Rosaria Greco, Antonina Stefania Mangione, Giorgio Sandrini, Giuseppe Nappi, Cristina Tassorelli

**Affiliations:** 1 Laboratory of Neurophysiology of Integrative Autonomic Systems, Headache Science Centre, “C. Mondino” National Neurological Institute, 27100 Pavia, Italy; 2 Department of Brain and Behaviour, University of Pavia, 27100 Pavia, Italy

**Keywords:** Migraine, Nitroglycerin, Hyperalgesia, CB2 agonist

## Abstract

**Background:**

Experimental animal models of migraine have suggested the existence of interactions between the endocannabinoid system and pain mediation in migraine. Extensive evidence has demonstrated a role for the cannabinoid-1 (CB1) receptor in antinociception. However, recent research suggests that also CB2 receptors, especially located outside the central nervous system, play a role in the perception of pain. Systemic administration of nitroglycerin (NTG) consistently induces spontaneous-like headache attacks in migraneurs; in the rat, systemic NTG induces a condition of hyperalgesia, probably through the activation of cerebral/spinal structures involved in nociceptive transmission. In this study we evaluated the role of CB2 receptors in two animal models of pain that may be relevant for migraine: the tail flick test and the formalin test performed during NTG-induced hyperalgesia.

**Methods:**

The study was performed in male Sprague-Dawley rats pre-treated with NTG (10 mg/kg, i.p.) or vehicle (4 hours before) and treated with the CB2 agonist AM1241 o dimethylsulfoxide (DMSO) 60 minutes before both the tail flick test and the formalin test.

**Results:**

AM1241 showed a significant analgesic effect in baseline conditions in both tests. Furthermore, when administered 3 hours after NTG administration, AM1241 at both doses significantly reduced the total number of flinches/shakes during phase II of the test.

**Conclusion:**

These findings suggest that the pharmacological manipulation of the CB_2_ receptor may represent a potential therapeutic tool for the treatment of migraine.

## Background

Experimental evidence suggests an important role for endocannabinoids in pain modulation. Administration of endocannabinoids, either systemically or directed at appropriate pain relay or modulatory sites, alters pain sensitivity and changes the processing of nociceptive information within discrete spinal and brain pathways. Therefore, the discovery of endocannabinoid system has prompted the development of a range of novel cannabinoid receptor agonists and antagonists, some of which show marked selectivity for CB_1_ or CB_2_ receptors. CB_1_ receptors are found in the central nervous system and have also been suggested to lie on peripheral nerve terminals [1]. Anandamide (AEA) is a full agonist at CB_1_ receptors and a partial agonist at CB_2_ receptors. Administration of AEA into the ipsilateral hindpaw of the rat reduced carrageenan-induced hyperalgesia [2] or formalin-induced nociception [3], indicating that activation of peripheral CB_1_ receptors and in part CB_2_ receptors, produces antinociception. CB_2_ receptors are mostly located outside of the central nervous system [4,5], and they were originally believed to be restricted to the periphery, primarily in the immune system, including mast cells, B and T cells, macrophages, and natural killer cells [6]. However, low levels of CB_2_ receptors were also observed in different CNS regions, with an upregulation in sites implicated in nociception [7,8]. Selective agonists for CB_2_ receptor have been shown to produce analgesic effects in preclinical models of inflammatory, neuropathic, and bone cancer pain [9,10]. Recent evidence shows that selective agonists of CB_2_ receptors reduce nociception in a variety of preclinical models without producing tolerance [11,12] or central side-effects [13], thus suggesting that CB_2_ agonists may represent an attractive therapeutic target for pain.

A deficiency of the endocannabinoid system has been postulated to underlie the pathophysiology of migraine, as suggested by clinical studies, although biochemical studies providing a scientific basis for the potential efficacy of endocannabinoids in migraine are so far really limited [14]. Using a well characterized animal model of migraine [15-18] based on the quantification of behavioral (nocifensive) and neurochemical changes induced by systemic nitroglycerin (NTG) - a vasodilator known to induce migraine-like headache in migraineurs [19-21] - we have gathered evidence to suggest a derangement of the endocannabinoid function in migraine. In this frame, NTG-induced hyperalgesia is associated with an increased activity of the enzymes involved in the catabolism of endocannabinoids in several brain areas and with an increased density of CB binding sites in the mesencephalon [22]. Additionally, AEA has proved to be effective in preventing both NTG-induced activation (c-Fos) of neurons in the nucleus trigeminalis caudalis (NTC) and NTG-induced hyperalgesia at the formalin test [23]. This model has been tested over the years with different drugs and is generally accepted as a reliable animal model of migraine [15-18,24]. Several lines of evidence suggest the existence of a condition of trigeminal sensitization in migraineurs, which results in hyperalgesia, allodynia, and cognitive dysfunction during and between episodes [25,26]. NTG is known to induce spontaneous-like headache attacks in migraine sufferers [16], probably as a consequence of sensitization phenomena [27]. In addition, NTG administration to migraineurs is associated to a significant facilitation in temporal summation of pain (reduced temporal summation threshold and increased painful sensation) when compared to baseline, to placebo condition and to controls. This finding suggests that migraineurs bear a susceptibility to develop migraine attack after NTG administration as a specific trait linked to a supersensitivity of the pain system to NTG [28]. AM1241 is a well characterized agonist of the CB_2_ receptor that mediates antinociception following systemic administration in animal models not specific for migraine [29]. The present study is aimed at evaluating the potential analgesic effect of AM1241 in animal models of hyperalgesia induced by NTG administration.

## Methods

Adult male Sprague-Dawley rats (weight 250-270 g, N = 4-8 for group) were evaluated in the present experiments. The principles of the Helsinki declaration and IASP’s guidelines for pain research in animals were rigorously applied [30]. The experimental research on animals was approved by ethics committee for research on animals of the University of Pavia (Document n. 2, 2012). Animals were housed in plastic boxes in groups of 3 with water and food available *ad libitum* and kept on a 12:12 hours light-dark cycle. All the rats were acclimatized to the test chamber before testing began.

### Drugs

AM1241 (Cayman Chemical) was injected intraperitoneally at two different doses: 2 and 4 mg/kg. The CB_2_ agonist was dissolved in 100% Dimethylsulfoxide (DMSO) as vehicle and administered in a volume of 1 ml/kg bodyweight [31] 60 minutes before the experimental tests, and 4 hours after NTG (or saline+alcohol +propylene glycol) administration. Nitroglycerin (NTG) (Astra Company, Italy), dissolved in saline, alcohol and propylene glycol, was injected i.p. at a dose of 10 mg/Kg.

For the Formalin test, a 100 μl volume of 1% formalin (formaldehyde diluted in 0.9% saline) was injected intraplantarly.

Rats were randomly divided into groups formed by 5-8 animals each, and underwent either the Tail flick test or the Formalin test. Rats were assigned to one of the treatment group according to a randomization list, whose codes were unblinded only after study completion. Therefore, the researchers who performed the behavioural testing (RG or SM) were blind to treatments.

### Tail flick test

Rats in this experiment were tested for latency of reflex tail withdrawal (Tail flick test, TFT) from a high intensity light beam, which was considered as a measure of physiological phasic pain.

The test was performed with a Tail Flick test instrument (Ugo Basile) that allowed automatic recording of tail-flick latency to radiant heat. Latency at each evaluation was calculated as the mean of three or five measurements in three different parts of the tail. A cut-off limit of exposure corresponding to 20 s was set to prevent tissue damage.

Each animal was placed on the recording platform of the instrument where it was kept under slight, painless restraint, with its tail positioned on the radiant heat window. The movement of the tail from window of the beam of light to hit a sensor was automatically registered.

### Formalin test

Rats in this experiment underwent Formalin test (FT) for the evaluation of inflammatory tonic pain.

One animal at a time was placed into a plexiglas observation chamber (10 × 20 × 24 cm) with a mirror (45° angle) positioned to permit unhindered observation of the animal paws. Formalin was injected subcutaneously into the center of the plantar surface of the left hind paw with slight restraint. A 26-gauge needle connected to a 1 ml syringe was used and the solution was delivered as rapidly as possible while the animal was immobilized. The rat was then replaced in the box, the clock was started and pain response was recorded for a period of 1 h [32].

The pain–related behaviour was quantified by counting the total number of flinches and shakes occurring for 1-min periods from 1 to 5 min (Phase I) and, then for 1-min periods at 5-min intervals during the period from 10 to 60 min (Phase II) after formalin injection. Phase I is generally considered the result of chemical activation of nociceptors, while Phase II reflects the inflammatory reaction and central processing.

Flinches/shakes were readily discriminated and were characterized as rapid and brief withdrawal movements or flexions of the injected paw.

### Experimental groups

**Figure 1:**
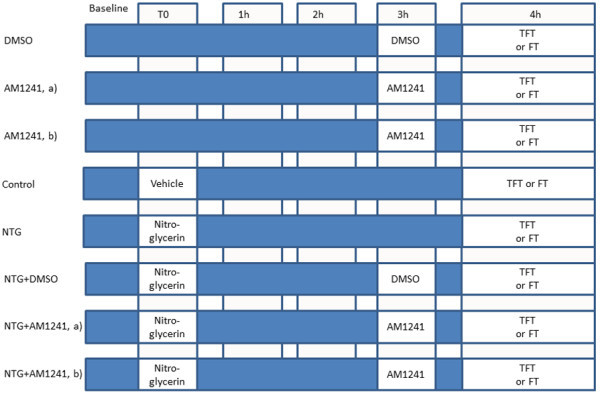
**Experimental plan and experimental groups.** TFT: tail flick test. FT: formalin test. DMSO Group: DMSO injected i.p. (2 ml /Kg, i.p.); AM1241 Group: AM1241 (a) (2 mg/kg, i.p.) dissolved in DMSO injected i.p.; AM1241 Group: AM1241 (b) (4 mg/kg, i.p.) dissolved in DMSO injected i.p.; Control Group: vehicle (saline, alcohol and propylene glycole) injected i.p. (3 ml/kg); NTG Group: nitroglycerin (dissolved in saline, alcohol and propylene glycole) injected i.p. at the dose of 10 mg/kg; NTG + DMSO Group: nitroglycerin (10 mg/kg) and DMSO (2 ml/kg) administered i.p.; NTG + AM1241 (a) Group: nitroglycerin (10 mg/kg, i.p.) and AM1241 (2 mg/kg, i.p.) administered i.p.; NTG + AM1241 (b) Group: nitroglycerin (10 mg/kg, i.p.) and AM1241 (4 mg/kg, i.p.) administered i.p.

The experimental plan was performed according to the treatment schedule indicated in Figure 1.

### Statistical evaluation

The effects of treatments upon the latency of the TFT were evaluated by means of the *Wilcoxon* rank-sum *test* (baseline vs. post-treatment). A probability level of less than 5% was regarded as significant. For FT, the total number of flinches/shakes evoked by formalin injection was counted separately for phase I and for phase II, as described above. Differences between groups were analysed by the one-way analysis of variance (ANOVA) followed by Tukey’s Multiple Comparison test. A probability level of less than 5% was regarded as significant.

## Results

### Tail flick test

**Figure 2:**
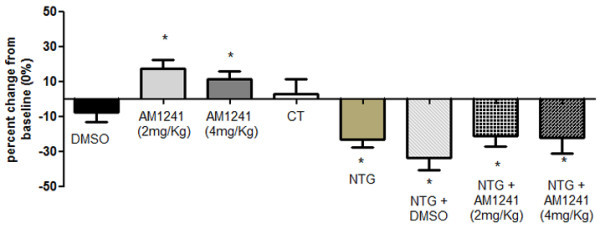
**Activity of AM1241 at the tail flick test in naïve rats and in rats treated with nitroglycerin.** Data are expressed as percent change (0%) from baseline ± SEM. In naïve rats, DMSO, used as vehicle, did not show significant effect on latency when compared with baseline levels, while AM1241 induced a significant increase of latency 60 minutes after its administration. Rats treated with nitroglycerin (NTG) showed hyperalgesia, demonstrated by the significant reduction in the latency of tail flicking 4 h after its administration when compared with baseline levels. The CT group did not show significant effect on latency when compared with baseline levels. Both doses of AM1241 failed to modify NTG-induced hyperalgesia. *p < 0.05 vs baseline.

The vehicle used for AM1241, DMSO, did not induce any significant effect on the tail flick latency (Figure 2). Conversely, AM1241 (2 and 4 mg/Kg) induced significant analgesia 60 minutes after its administration when compared to baseline (Figure 2). NTG, either alone or in association with DMSO, induced a hyperalgesic response at the TFT as suggested by the significant decrease in the latency of the tail flick response 4 hours after its administration when compared to control group (CT). Administration of AM1241 did not counteract NTG-induced hyperalgesia.

### Formalin test

**Figure 3:**
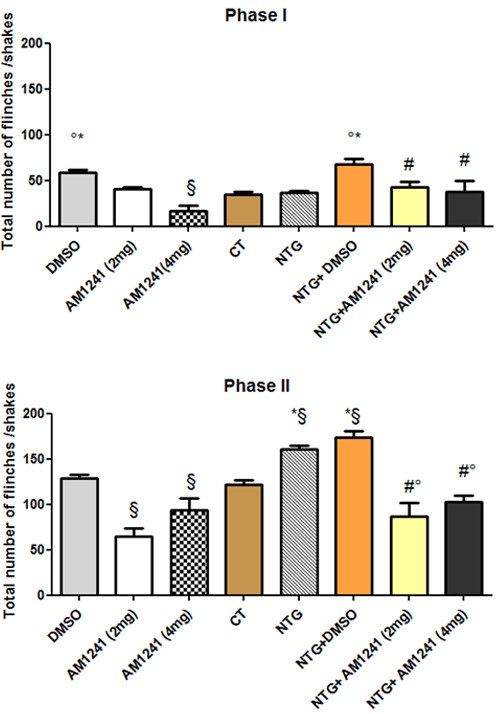
**Activity of AM1241 at the formalin test in naïve rats and in rats treated with nitroglycerin.** In the vehicle groups (DMSO and Control, CT), the injection of formalin resulted in a highly reliable, typical, biphasic pattern of flinches/shakes of the injected paw, being characterized by an initial acute phase of nociception within the first 5 min, followed by a prolonged tonic response from 15 to 60 min after formalin injection. DMSO administration induced a significant increase in the nocifensive behaviour of animals only in phase I, when compared with the CT group. No difference was reported between CT and DMSO groups in phase II. NTG administration, alone or with DMSO, significantly increased the total number of flinches/shakes in phase II of formalin test, when compared either to CT group or to DMSO group. In naive rats, the lower dose of AM1241 (2 mg/kg) significantly inhibited nocifensive behaviour induced by formalin injection during phase I, while the higher dose (4 mg/Kg) inhibited the nocifensive behaviour in both phases of the test when compared with DMSO group. In rats treated with nitroglycerin (NTG), both doses of AM1241 significantly reduced the total number of flinches/shakes in phase I of test, when compared to NTG + DMSO group, and phase II of the test when compared to both NTG and NTG + DMSO groups. Data are expressed as mean ± SEM. ANOVA analysis followed by Tukey’s Multiple Comparison test, *p < 0.05 vs CT; °p < 0.05 vs NTG; #p < 0.05 NTG + DMSO; §p < 0.05 vs DMSO.

In the two vehicle groups (DMSO and CT), the injection of formalin resulted in a highly reliable, typical, biphasic pattern of flinches/shakes of the injected paw, being characterized by an initial acute phase of nociception within the first 5 min, followed by a prolonged tonic response from 15 to 60 min after formalin injection. DMSO administration induced a significant increase in the nociceptive behaviour of animals in phase I of the test, when compared with the CT group (Figure 3), while no difference was reported between CT and DMSO groups as regards phase II. NTG administration, alone or with DMSO, significantly increased the total number of flinches/shakes in phase II of formalin test, when compared either to control group (CT) or to DMSO group (Figure 3). The lower dose of AM1241 (2 mg/Kg) significantly inhibited nociceptive behaviour induced by formalin injection only in phase II when compared to the DMSO group. The higher dose (4 mg/Kg) of the CB_2_ agonist significantly inhibited nociceptive behaviour induced by formalin injection during both phases of the test when compared with DMSO group. Both doses of AM1241 proved effective in counteracting NTG-induced hyperalgesia in phase II, as suggested by the reduction of the nocifensive behaviour of animal pre-treated with NTG. Comparison of the 2 mg and 4 mg AM1241 groups with the NTG + AM1241 (2 mg and 4 mg) groups did not show any significant differences in phase II of the test.

## Discussion

Several lines of evidence have highlighted the importance of cannabinoid-mediated analgesia for nociceptive processing. Exogenous cannabinoids reduce responsiveness to noxious thermal, chemical and mechanical stimuli in rats. Cannabinoids induce antinociception by acting in neuroanatomical regions subserving transmission and modulation of pain signals, including, the periaqueductal gray (PAG) [33,34] and the basolateral nucleus of amygdala [35]. The analgesic effect of endocannabinoids can be attributed in part to a neuronal mechanism based on the activation of CB_1_ receptors expressed in primary afferent neurons. Indeed, it has been clearly demonstrated that CB_1_ receptors are involved in modulation of pain signals via the inhibition of the release of neurotransmitters such as γ-aminobutyric acid (GABA), glutamate, dopamine, noradrenaline and acetylcholine [36]. Also CB_2_ receptors appear to contribute to the analgesic effect, as suggested by the attenuation of pain in animal models of inflammatory and nociceptive pain [37]. Originally described in immune cells [4], CB_2_ receptors have been demonstrated in human peripheral nerves after injury, as well as in brain regions, i.e. brainstem, which are particularly relevant for nociceptive integration [38-41].

Endocannabinoid deficit may be involved in the pathophysiology of migraine as supported by an increasing body of evidence [22,23]. In previous studies, using the same animal model proposed in the present study, we have provided data supporting the role of endocannabinoids in NTG-induced hyperalgesia [22]. In particular, we have demonstrated that NTG injection causes specific changes in endocannabinoids content in discrete cerebral areas, while the administration of AEA (20 mg kg^−1,^ i.p.), a CB_1_/CB_2_ agonist, reduces NTG-induced c-Fos expression in the NTC [22].

The analgesic effects derived from selective stimulation of CB_1_ receptors is well known, but the relevant side-effects related to the stimulation of CB_1_ receptors [11] have limited the clinical development of this therapeutic line. Interestingly, CB_2_ agonists can reduce nociception in several preclinical models of pain without producing tolerance [9] or central side-effects [11]. Therefore CB2 antagonists seems more attractive as potential targets for modulating pain.

In the present study, we have shown that activation of CB_2_ receptor, by means of AM1241 administration, induces analgesia at both the tail flick and the formalin tests. These findings are in agreement with behavioural, electrophysiological and neurochemical studies that showed the role for CB_2_ receptor activation in modulating inflammatory nociception [40,42-44].

Furthermore, we detected an anti-hyperalgesic effect of AM1241 when the experimental paradigm was represented by phase II of formalin test in NTG treated animals, i.e. a condition of hyperalgesia mediated by the peripheral release of inflammation mediators and by central modulation.

The formalin test adopted in this study is based on the stimulation of sensorial areas that are outside of the trigeminal distribution, which may limit the applicability of our findings to migraine. However the model seems quite specific for cephalic pain since we have previously demonstrated that plantar injection of formalin in rats induces significant changes in calcitonin gene-related peptide (CGRP) immunoreactivity in the superficial laminae I and II ipsilateral to the injection side also in the NTC and that systemic NTG administration causes a reduction in CGRP-imunoreactivity in the NTC, but not in the lumbar dorsal horns [45]. Taken together with the demonstrated capability of NTG to induce spontaneous-like migraine attacks in migraineurs, these findings suggest that NTG-potentiated formalin test is a relevant model for investigating migraine circuitry. Though so far unexplained, the observation that a nociceptive stimulus delivered at the paw level is associated with NTC activation is further reported by the study of Han et al., [46], which showed that formalin injection in the paw induces Fos expression in NTC, and in other brainstem areas (i.e locus coeruleus) known to be involved in the modulation of migraine pain [47]. The possible mechanisms underlying NTG-induced hyperalgesia are presently elusive. However, it is becoming increasingly evident that NTG exerts its hyperalgesic effect through central and peripheral mechanisms [15,16]. NTG may induce indeed a direct hyperalgesic effect via the formation of nitric oxide (NO) and via CGRP release in the NTC [42,48], or indirectly via the activation of NOS synthesis at the meningeal level as a consequence of a sensitization of the trigeminovascular system [49-51].

CB_2_ receptors are expressed predominantly, but not exclusively, outside the CNS [41,42], where they are localized extensively to cells of the immune system [52]. CB_2_ receptors have been detected in cultured DRG neurons and in afferent fibers in the dorsal horn of the spinal cord [53], which confirms that CB_2_ receptors are present in an area that is relevant for the mediation of the response to the formalin test.

In our experimental paradigm, CB_2_ receptor activation may interfere with the mechanisms associated with NTG-induced hyperalgesia at two levels: central and peripheral. As regards the central one, Beltramo et al., [40] demonstrated that AM1241 reduces capsaicin-induced CGRP release in dorsal root ganglia (DRG), confirming that CB_2_ activation may elicit cause analgesia by acting not only at non-neuronal peripheral sites but also at the neural level. Accordingly, the activation of CB_2_ receptors reduces spinal fos protein expression and pain behaviour in a rat model of inflammation [42]. Taken together, these observations suggest that one possible mechanism through which AM1241 reduces NTG-induced hyperalgesia is the inhibition of CGRP release in laminae I and II of the NTC (lower brainstem and cervical spinal cord) [45], although further studies are needed to confirm this hypothesis. Indeed, following NTG administration, CGRP immunoreactivity decreased steadily in the NTC, whereas substance P immunoreactivity increased transiently [45].

When considering the potential peripheral mechanisms underlying the inhibitory effect of CB_2_ agonism upon NTG-induced hyperalgesia, it seems noteworthy that emerging literature implicates a role for neuroimmune interactions in contributing to the development or maintenance of pathological pain states [54], and that it has been shown that NTG administration causes a delayed meningeal inflammation, as showed by activation of inducible NO synthases (iNOS) in macrophages of rodents, and a prolonged cold allodynia and heat hyperalgesia with a time-course consistent with NTG-induced migraine attacks [49,55]. Therefore, we suggest that another possible site of action for AM1241 is represented by the dura, where the CB_2_ agonist may counteract NO-induced activation of macrophages via the inhibition of NO by iNOS. Indeed, activation of CB2 receptors on non-neuronal cells has been postulated to suppress the release of inflammatory mediators that sensitize nociceptors [56]. This hypothesis is partially supported by the demonstrated interactions between the endocannabinoid and the nitrergic systems in CB_2_ signalling. The activation of the CB_2_ receptor seems indeed to be associated with a reduced expression of iNOS [57]. Additionally, CB_2_ stimulation seems to suppress the release of pro-inflammatory factors such as NO and tumor necrosis factor alpha (TNF-α) in macrophages [58].

A collateral finding of this study is represented by the increase in nocifensive behavior in phase I of the formalin test following the administration of DMSO. This finding is partially in agreement with the observations of Colucci et al. (2008) [59] who showed an increase in the nocifensive behavior in both phases of the formalin test when DMSO was applied subcutaneously in the mouse paw. Conversely, the same study demonstrated an anti-nociceptive activity when DMSO was administered centrally or intraperitoneally [59]. These results suggest that DMSO displayed opposite effects on nociception and inflammation, depending on the route of administration. DMSO is one of the most common solvents used experimentally to dissolve hydrophobic substances for *in vivo* and *in vitro* purposes. The exact mechanism underlying DMSO pro-nociceptive activity is not known, but our findings show that the pro-nociceptive effect observed in phase I of the formalin test in the DMSO and NTG + DMSO groups (Figure 3) was counteracted by activation of CB2 receptors.

## Conclusions

The present study lends further support to the therapeutic potential in migraine of probes that interfere with the endocannabinoid system. More specifically, stimulation of CB_2_ receptors seems promising as it counteracts NTG-induced hyperalgesia in phase II of the formalin test and it is theoretically less likely to induce CNS side effects. However, the impact of long term treatment with CB2 agonists on their anti-hyperalgesic efficacy and on their effect on the immune system function remains to be elucidated.

## Abbreviations

CB1 receptor: Cannabinoid-1 (CB_1_) receptor; CB2 receptor: Cannabinoid-2 (CB_2_) receptor; NTG: Nitroglycerin; DMSO: Dimethylsulfoxide; CNS: Central nervous system; AEA: Anandamide; NTC: Nucleus trigeminalis caudalis; DRG: Dorsal root ganglia; PAG: Periaqueductal gray; GABA: γ-aminobutyric acid; DRG: Dorsal root ganglia; CGRP: Calcitonin gene-related peptide; NO: Nitric oxide; iNOS: Inducible nitric oxide synthases; TNF-α: Tumor necrosis factor alpha; TFT: Tail flick test; FT: Formalin test.

## Competing interests

The authors declare to have no competing interests.

## Authors’ contributions

RG instructed the experiments, ASM performed the experiments. RG analysed the data and drafted the manuscript. CT revised the manuscript. All authors contributed to the idea of the study, and read and approved the final manuscript.
